# Comment on the article, “Trauma Care Systems in Saudi Arabia: An Agenda for Action”

**DOI:** 10.4103/0256-4947.65258

**Published:** 2010

**Authors:** Kenneth L. Mattox

**Affiliations:** Distinguished Service Professor, Baylor College of Medicine Houston

**To the Editor:** I was in Riyadh at the King Faisal Specialist Hospital and Research Centre as senior surgeon with the Baylor Heart Team, 30 years ago when this journal had its beginning. I knew the first editor Dr. Nizar Feteih, a brilliant cardiologist and his thoracic surgeon brother, Dr. Weil Feteih, who we had the honor to provide some of his training in Houston. I congratulate you and your editorial board, as well as the visionary board members who have brought this high impact medical journal so very far. The integration of biomedical research, postgraduate courses, basic medical education, residency training programs, superb medical journals, and integrated high quality medical care has made medical care in Saudi Arabia an ever increasing foundation of professional excellence and creating standards for integrated collaborative systems which are models for many countries in the world. Congratulations, for this growth and excellence is reflected in the appearance of your journal and the quality of its articles.

Leadership in medicine, surgery, publications, astronomy, chemistry, alchemy, medical education, and research are not new to the Arabian Peninsula or the Islamic Middle East. I was able to give a Key Note Opening Address to the recent medical symposium in Doha, Qatar on the “Contributions of ibn Nafis to the Advancement of Medicine and Trauma.” He was but one of many pacesetters in the medical sciences who were far ahead of their European colleagues in advancing knowledge and care. Your journal continues to publish the legacy of your medical historic past and your contributions to the present improvement in health care, and your visions for the future. With more than 50% of your current population below the age of 20, your challenges are well founded and exciting.

I could not miss, nor should I ignore to mention, the excellent and pointed article in the first issue of Volume 30, entitled, “Trauma Care Systems in Saudi Arabia: An Agenda for Action, ” by Mohammed Y. Al-Naami, Maria A. Arafah, and Fatimah S. Al-Ibrahim.^1^ No better article exists describing the systems approach to the many components of Trauma Care, and the article speaks for itself. I have a moral obligation to comment on a number of facts cited in this fantastic article. The leading cause of death in Saudi Arabia is TRAUMA. The infrastructure to address this public health issue is inadequate and incomplete, at all levels of the system, as documented by this article: too few ambulances, too few ambulance attendants, too little integration of communications, too few trauma centers, too little support for this health issue as compared to more “lucrative” and technically attractive disciplines, such as obstetrics, cancer care, heart surgery, transplantation, and others. The details of this article indicate that the elements for success are well known, and the reference list documents the root source of the supporting information. Few trauma centers of excellence exist throughout the Middle East, with nests of growing expertise and interest in Riyadh, Doha, Dubai, and Abu Dhabi. An emergency room is different from a Trauma Center, and multiple supporting elements are required in order for the trauma system to function as an integrated collaborative network. The internet is the best example of such a network, and it functions to a much higher level than trauma care throughout the Middle East.

For 30 years, your journal has documented the tremendous advancement you have made in providing advanced medical care to your citizens and visitors. Now your journal has published a detailed knowledge of a trauma system, cited the leading cause of death in Saudi Arabia, and outlined a systematic approach to integrating the elements to make the best trauma network in the world. Congratulations on your continuing leadership and education.
